# Polymerized-Type I Collagen Induces Upregulation of Foxp3-Expressing CD4 Regulatory T Cells and Downregulation of IL-17-Producing CD4^+^ T Cells (Th17) Cells in Collagen-Induced Arthritis

**DOI:** 10.1155/2012/618608

**Published:** 2011-10-19

**Authors:** Janette Furuzawa-Carballeda, Perla Macip-Rodríguez, Angeles S. Galindo-Feria, David Cruz-Robles, Virgina Soto-Abraham, Sergio Escobar-Hernández, Diana Aguilar, Deshiré Alpizar-Rodríguez, Karen Férez-Blando, Luis Llorente

**Affiliations:** ^1^Department of Immunology and Rheumatology, Instituto Nacional de Ciencias Médicas y Nutrición Salvador Zubirán, Vasco de Quiroga No. 15, Colonia Sección XVI, Tlalpan, 14000 Mexico City, DF, Mexico; ^2^Department of Pathology, Instituto Nacional de Cardiología Ignacio Chávez, Juan Badiano No. 1, Colonia Sección XVI, Tlalpan, 14080 Mexico City, DF, Mexico; ^3^Department of Experimental Pathology, Instituto Nacional de Ciencias Médicas y Nutrición Salvador Zubirán, Vasco de Quiroga No. 15, Colonia Sección XVI, Tlalpan, 14000 Mexico City, DF, Mexico

## Abstract

Previous studies showed that polymerized-type I collagen (polymerized collagen) exhibits potent immunoregulatory properties. This work evaluated the effect of intramuscular administration of polymerized collagen in early and established collagen-induced arthritis (CIA) in mice and analyzed changes in Th subsets following therapy. Incidence of CIA was of 100% in mice challenged with type II collagen. Clinimorphometric analysis showed a downregulation of inflammation after administration of all treatments (*P* < 0.05). Histological analysis showed that the CIA-mice group had extensive bone erosion, pannus and severe focal inflammatory infiltrates. In contrast, there was a remarkable reduction in the severity of arthritis in mice under polymerized collagen, methotrexate or methotrexate/polymerized collagen treatment. Polymerized Collagen but not methotrexate induced tissue joint regeneration. Polymerized Collagen and methotrexate/polymerized collagen but not methotrexate alone induces downregulation of CD4^+^/IL17A^+^ T cells and upregulation of Tregs and CD4^+^/IFN-*γ* ^+^ T cells. Thus, Polymerized Collagen could be an effective therapeutic agent in early and established rheumatoid arthritis by exerting downregulation of autoimmune inflammation.

## 1. Introduction

Rheumatoid arthritis (RA) is a common systemic disorder characterized by autoimmunity and chronic inflammation of multiple joints. Collagen-induced arthritis (CIA) is a well-established animal model of RA [1]. CIA is induced in genetically susceptible strains of mice by immunization with type II bovine collagen (CII). Although the effector mechanisms of inflammation ultimately result in pathogenic lesions of joints (inflammatory component of CIA), there is considerable evidence implicating CII-specific CD4^+^ T cells as primary mediators of disease induction (T-cell immunity component of CIA) [2, 3]. After antigenic stimulation naive CD4^+^ T cells develop into different types of helper T cells, each produces its own set of cytokines that mediate different responses in CIA. It has been well documented that Th1 cells produce interferon (IFN)-*γ* and interleukin (IL)-2 and have been considered to be the major mediator of the disease [4, 5]. However, the notion that CIA is a Th1-mediated disorder has been challenged by studies using Th1-defective mice [6]. Mice lacking IFN-*γ*, IFN-*γ* receptor, or IL-12p35 develop accelerated arthritis after induction of CIA [7, 8]. Furthermore, recent studies have suggested that highly proinflammatory IL-17-producing Th17 cells, rather than Th1 cells, are central to the pathology of autoimmune arthritis [9, 10]. IL-17-producing CD4^+^ T cells contribute to severe synovitis, pannus formation, joint destruction in arthritis joints and autoimmune inflammation [6]. On the other hand, there is ample evidence that CD4^+^/CD25^+^/Foxp3^+^ regulatory T (Treg) cells also play a critical role on the inhibition of autoimmune reaction. Thus, a reciprocal relationship between the differentiation of Th17 and Treg cells has been reported [11]. Hence, therapeutic strategies of RA should consider the regulation of Th17 and Treg differentiation as well as the inhibition of proinflammatory cells and Th1 cytokines.

In order to shift the cytokine balance to anti-inflammatory cytokines in RA and to delete hyperactive proinflammatory Th17 and Th1 cells that closely associate with etiology of RA, it is of utmost importance to discover novel biological substances that can selectively suppress the function or downregulate activated Th1 and Th17 cells, whilst at the same time enhance Treg cell function. 

This study focuses on the effect of polymerized-type I collagen on Th subsets and its primary mechanism of action in early and established CIA in mice. Polymerized Collagen is a *γ*-irradiated mixture of atelopeptidic porcine type I collagen and polyvinylpyrrolidone, which has immunomodulatory properties [12–14]. One percent Polymerized Collagen addition to synovial tissue cultures from non-RA and RA cultures does not induce any change in DNA concentration or metabolism. However, the addition of the biodrug to RA synovial tissue cultures modifies the histological and biochemical pattern of fibrosis, without changing the total collagen content. Polymerized Collagen induces the recovery of type III collagen at similar levels to those detected in normal synovial tissue. The biodrug diminishes the accumulation of dense and tightly packed type I collagen fibers and contributes to establish similar tissue architecture to that observed in normal synovium. Polymerized Collagen induces a decrease of collagenolytic activity, mainly calcium-independent collagenase activity (cathepsins) and the increase of TIMP-1, as well as type III collagen production. The chronic inflammatory process is altered by Polymerized Collagen action, presumably due to the downregulation of IL-1*β* and TNF-*α*, ELAM-1, VCAM-1, ICAM-1, and Cox-1 [15, 16]. 

Subcutaneous or intramuscular administration of Polymerized Collagen in combination with methotrexate to RA patients was safe and well tolerated in the treatment of this pathology [17]. The biodrug induced a statistically significant clinical improvement in basal versus 3- or 6-month treatment [18]. Besides, intramuscular administration diminished C-reactive protein (CRP) and rheumatoid factor (RF) levels, and patients required lower doses of methotrexate versus placebo. Thirty percent achieved remission. No differences in serological or hematological variables were found. Adverse events were not detected, except pain lasting <5 min at the injection site [18].

In this study, we show that Polymerized Collagen suppresses the development of CIA by enhancing a proportion of Treg cells while simultaneously diminishing Th17 cells suggesting that Polymerized Collagen ameliorates autoimmune arthritis through the regulation of both Treg/Th17 differentiation and Th1/Th2 balance. 

## 2. Materials and Methods

### 2.1. Mice

Male DBA1/J mice (7-8 weeks old, 20–22 g) were obtained from Harlan S.A. de C.V. (Mexico City, Mexico). Mice were kept under specific pathogen-free conditions. All animals were set aside under standard conditions in a 12 h day/night rhythm with access to food and water *ad libitum*. All animal procedures and experiments were carried according to international guidelines (ICLAS-WHO) and national law (NOM 062-ZOO-1999). The protocol of this study was approved by the Animal Care and Research Committee of the Instituto Nacional de Ciencias Médicas y Nutrición Salvador Zubirán. 

### 2.2. Induction of CIA

Arthritis was induced according to methods as described previously [1]. Chicken CII (SIGMA-Aldrich Co. St. Louis, MO, USA) was dissolved in 0.05 M acetic acid to a concentration of 2 mg/mL at 4°C, and emulsified with an equal volume of complete Freund's adjuvant (SIGMA-Aldrich Co.) Male mice were injected intradermally at the base of the tail with 0.1 mL of emulsion containing 100 *μ*g of chicken CII. Twenty-one days after the primary immunization, mice were boosted with 0.1 mL of the mixture of 2 mg/mL chicken CII and incomplete Freund's adjuvant (SIGMA-Aldrich Co.) via the same route. In each experiment, a control group mice were injected with citric/citrate buffer alone.

### 2.3. Evaluation of Arthritis Severity in Mice with CIA

The severity of arthritis was measured by scoring each limb from 0 to 4 grades and by summing up the scores of four limbs: 0 = normal; 1 = erythema or swelling of one or several digits; 2 = erythema and moderate swelling extending from the ankle to the mid-foot (tarsals); 3 = erythema  and severe swelling extending from the ankle to the metatarsal joints; 4 = complete  erythema and swelling encompassing the ankle, foot, and digits, resulting in deformity and/or ankylosis. The maximum score for each animal is thus 16 [3]. Paw edema in each animal was measured with a vernier (Mitutoyo America Co.). All the evaluations were made by the same observer.

### 2.4. Disease Models

#### 2.4.1. Toxicity

Sixteen mice (*n* = 4 per group) without arthritis were treated by intramuscular administration once weekly during six weeks with 100 *μ*L of (a) placebo (citric/citrate buffer), (b) polymerized-type I collagen (0.17 mg of collagen), (c) Methotrexate (2.5 mg/kg) [19], or (d) methotrexate (2.5 mg/kg)/Polymerized-type I collagen (0.17 mg of collagen). Two mice of each group were sacrificed at the 6th and 13th weeks.

#### 2.4.2. Early Arthritis

Forty-eight male mice (*n* = 12 per group) were injected with emulsion of chicken CII. Twenty one days after the primary immunization, mice were boosted. At the same time, mice were treated with 100 *μ*L of (a) placebo (citric/citrate buffer), (b) Polymerized-Type I Collagen, (c) methotrexate (2.5 mg/kg) or (d) methotrexate/Polymerized-Type I Collagen. Six mice of each group were sacrificed at the 6th and 13th weeks.

#### 2.4.3. Established Arthritis

Forty eight male mice (*n* = 12 per group) were injected with emulsion of chicken CII. Twenty one days after the primary immunization, mice were boosted. One week later, mice were treated with 100 *μ*L of (a) placebo (citric/citrate buffer), (b) Polymerized-Type I Collagen, (c) methotrexate, or (d) Methotrexate/Polymerized-Type I Collagen. Six mice of each group were sacrificed at the 8th and 13th weeks.

### 2.5. Drug Treatment

One hundred *μ*L of (a) placebo (citric/citrate buffer), (b) Polymerized-Type I Collagen, (c) methotrexate (2.5 mg/kg), or (d) methotrexate/polymerized-type I collagen was intramuscularly administered once weekly during six weeks.

### 2.6. Histological Analysis

On day 35 and day 98, the mice were killed in a carbon dioxide chamber, and the hind paws were collected, fixed with 10% buffered formalin, and then decalcified in 5% formic acid and embedded in paraffin. Sections (5 *μ*m) of whole hind paws were stained with hematoxylin and eosin and PAS technique. Histopathological changes were scored by a blinded observer using the previously reported parameters: 0 = normal joint structure; 1 = mild changes, synovitis and pannus front with view discrete cartilage focal erosions; 2 = moderate changes, accompanying loss of large areas of cartilage, eroding pannus front and synovial hyperplasia with infiltrating mononuclear cells and polymorphonuclear cells; 3 = severe synovitis, cartilage, and bone erosion; 4 = total destruction of joint architecture [20].

### 2.7. Cell Preparations and Flow Cytometric Analysis

Spleens were isolated from mice. Splenocytes were stained with 5 *μ*L of anti-CD4-FITC-labelled monoclonal antibody (BD Biosciences, San Jose, Calif, USA) at room temperature in the dark for 20 min. After two washes, cells were permeabilized with 200 *μ*L of cytofix/cytoperm solution (BD Biosciences) at 4°C for 20 min. After two washes with permwash solution (BD Biosciences), cells were stained for intracellular cytokines and transcription factors with 5 *μ*L of anti-IL-17A-PE-labelled, anti-IFN-*γ*-PE-labeled anti-IL-4-PE-labelled, and PE-labelled anti-Foxp3 for Tregs (BD Biosciences), for 30 min at 4°C in the dark. Finally, after washing with permwash solution, splenocytes were analyzed by flow cytometry with a FACScan (BD Biosciences). A total of 10000 events were recorded for each sample and analyzed with the CellQuest software (BD Biosciences). Results are expressed as the relative percentage of IL-17A, IFN-*γ*, IL-4, or Foxp3-expressing cells in each gate. In order to avoid false positive PE results and also for setting compensation for multi-color flow cytometric analysis, we performed instrument calibration/standardization procedures each day according to established protocols of our laboratory. Briefly, we run an unstained (autofluorescence control) and permeabilized PBMCs sample. Autofluorescence control (unstained cells) was compared with single-stained cell-positive controls to confirm that the stained cells were on scale for each parameter. Besides, BD Calibrite 3 beads were used to adjust instrument settings, set fluorescence compensation, and check instrument sensitivity (BD CaliBRITE, BD Biosciences).

### 2.8. NF*κ*B/I*κ*B*α* Flow Cytometric Analysis

Splenocytes were permeabilized with 200 *μ*L of cytofix/cytoperm solution (BD Biosciences) at 4°C for 20 min. After two washes with permwash solution (BD Biosciences), cells were stained for intracellular transcription factors with 5 *μ*L of anti-NF*κ*Bp65-FITC-labelled monoclonal antibody (Santa Cruz, Calif, USA) and 5 *μ*L of anti-I*κ*B*α* PE-labeled monoclonal antibody (I*κ*B*α* binds to the p65 subunit of p50–p65 heterocomplex through ankyrin repeats) (Santa Cruz), for 30 min at 4°C in the dark. After washing with permwash solution, splenocytes were analyzed by flow cytometry with a FACScan (BD Biosciences). A total of 10000 events were recorded for each sample and analyzed with the CellQuest software (BD Biosciences). Results are expressed as the relative percentage of NF*κ*Bp65^+^/I*κ*B*α* ^+^ cells in each gate. 

### 2.9. Statistical Analysis

Statistical analysis was performed using the SigmaStat11 program by one way analysis of variance on Ranks and by Holm-Sidak method for all pairwise multiple comparison procedures. Data were expressed as the mean ± SEM. The *P-*values smaller than or equal to 0.05 were considered as significant.

## 3. Results

### 3.1. Effect of Polymerized-Type I Collagen on Toxicity Model

Treatments evaluated in this study have no *in vivo* toxicity for mice. It was evaluated by macroscopical and histopathological analysis of kidneys, heart, lungs, spleen, lymph nodes, and hind paws. All tissues resembled normal architecture. No inflammatory infiltrates or other abnormalities were observed (*data not shown*).

### 3.2. Effect of Polymerized-Type I Collagen on Attenuation of Early and Established Arthritis Model

**Figure 1:**
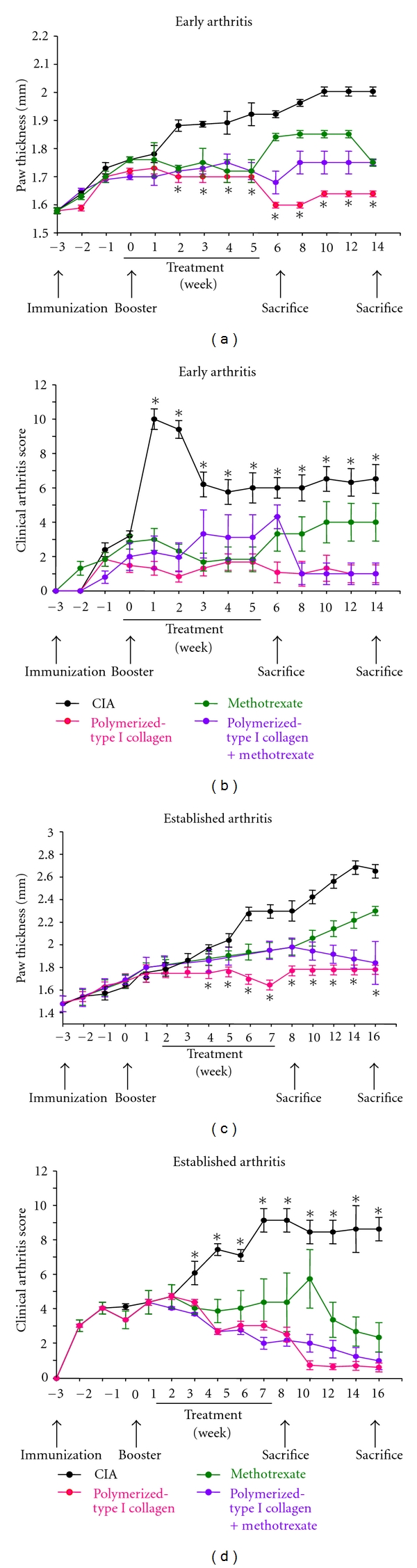
Preventive and therapeutic effects of polymerized-type I collagen on mouse CIA. (a) Paw thickness in early arthritis model. One hundred microliters of Polymerized Collagen, 100 *μ*L of Polymerized Collagen, and 100 *μ*L of methotrexate (2.5 mg/kg) or 100 *μ*L of methotrexate (2.5 mg/kg)/Polymerized Collagen were administered intramuscular once a week during 6 weeks at the same time of that of the booster. Citric/citrate buffer was injected as a vehicle control. Data represent mean ± SEM (each group, *n* = 6). (b) Clinical arthritis score in early arthritis model (*n* = 6). (c) Paw thickness in established arthritis model. One hundred microliters of Polymerized Collagen, 100 *μ*L of Polymerized Collagen, and 100 *μ*L of methotrexate (2.5 mg/kg) or 100 *μ*L of methotrexate (2.5 mg/kg)/Polymerized Collagen were administered intramuscularly once a week during 6 weeks. Treatments were started on 14 days after the booster. Citric/citrate buffer was injected as a vehicle control. Data represent mean ± SEM (each group, *n* = 6). (d) Clinical arthritis score in established arthritis model (*n* = 6). *P < 0.05.

To investigate the effect of polymerized-type I collagen in the progression of CIA, intramuscular administration of biodrug was initiated one week before booster of CII. The dose selected was based on our previous *in vitro* and *in vivo* results [16, 17]. Typically, DBA1/J mice developed signs of arthritis after the second immunization given at 21 day after the first CII immunization and showed maximum arthritis around day 35–42 (Figure 1). Mice of early arthritis model treated with polymerized-type I collagen showed significant reductions in the severity of CIA compared with placebo (Figures 1(a) and 1(b)). On day 8–16, arthritis score and paw thickness reached their peaks. Polymerized Collagen and methotrexate/Polymerized Collagen treatments more than methotrexate alone significantly suppressed arthritis severity scores. A similar pattern of response was observed on established arthritis model (Figures 1(c) and 1(d)). Paw thickness was normal in early and established CIA models in groups treated with Polymerized Collagen and methotrexate/Polymerized Collagen (Figures 1(a) and 1(c)). However, methotrexate treatment does not diminish edema and induces fever (∆≈1.5°C).

### 3.3. Histopathological Analysis of the Polymerized-Type I Collagen Effect on Early and Established Arthritis Model

**Figure 2:**
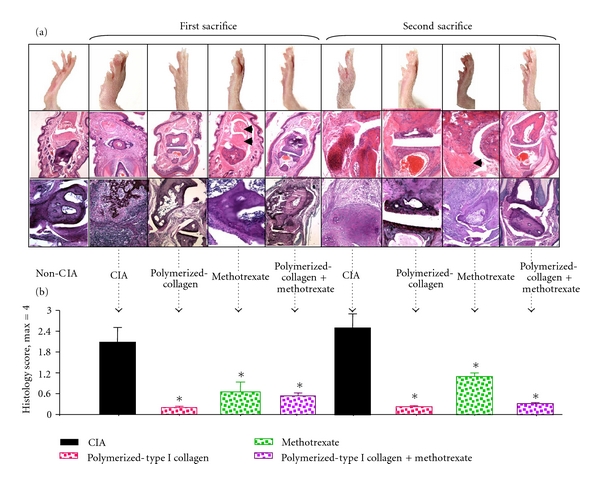
Preventive effect of polymerized-type I collagen on the histological damage in CIA mice. (a) Representative section of joint histopathology is shown. Upper panel: hematoxylin and eosin stained and lower panel: PAS stained; magnification: 10x. Arrow heads point out nodules. (b) Pathology scores of each group were calculated and expressed as mean ± SEM (*n* = 6). *P < 0.05.

Histopathological analysis was performed on hind paws of CIA mice harvested on day 35 and 98 after booster immunization by two blinded investigators. Representative images of hematoxylin and eosin-stained and PAS-stained joint tissue sections from the groups treated with placebo, Polymerized-Type I Collagen, methotrexate, and methotrexate/polymerized-type I collagen are presented in Figure 2. Mice showed typical arthritis, which is characterized by extensive infiltration of inflammatory cells, synovial hyperplasia, and bone erosion. Treatment with polymerized-type I collagen showed no signs of inflammation. Whereas methotrexate/polymerized-type I collagen resulted in significant reduction in cellular infiltration, pannus formation, and destruction of cartilage and bone in arthritic joints with a low pathogenic score of CIA mice in both models. Methotrexate induced other tissue abnormalities such as the presence of nodules (amorphous fibrin tissue like) and a low quality wound repair tissue as well as inflammatory infiltrates. The effect of polymerized-type I collagen and methotrexate/polymerized-type I collagen was sustained until the second sacrifice. Thus, histological evaluations confirmed the characteristic arthritic lesions and showed an excellent correlation with clinical grading (Figure 2).

### 3.4. Polymerized-Type I Collagen Modulates Th Spleen Cell Population on Early and Established CIA Model

**Figure 3:**
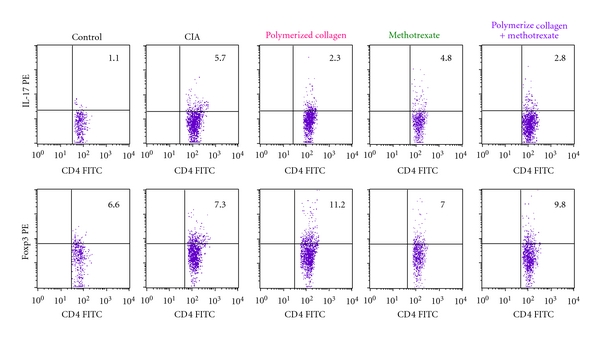
Representative flow plots of IL-17A- and Foxp3-expressing CD4^+^ T cells.

To investigate Th cytokine patterns, mice were sacrificed by a blinded researcher at two different periods after booster immunization, on day 35 and 98. One control mice group without CIA was included. Cell suspensions from spleens were obtained and analyzed for *ex vivo *cytokine production by intracellular staining using flow cytometry (Figure 3). 

**Figure 4:**
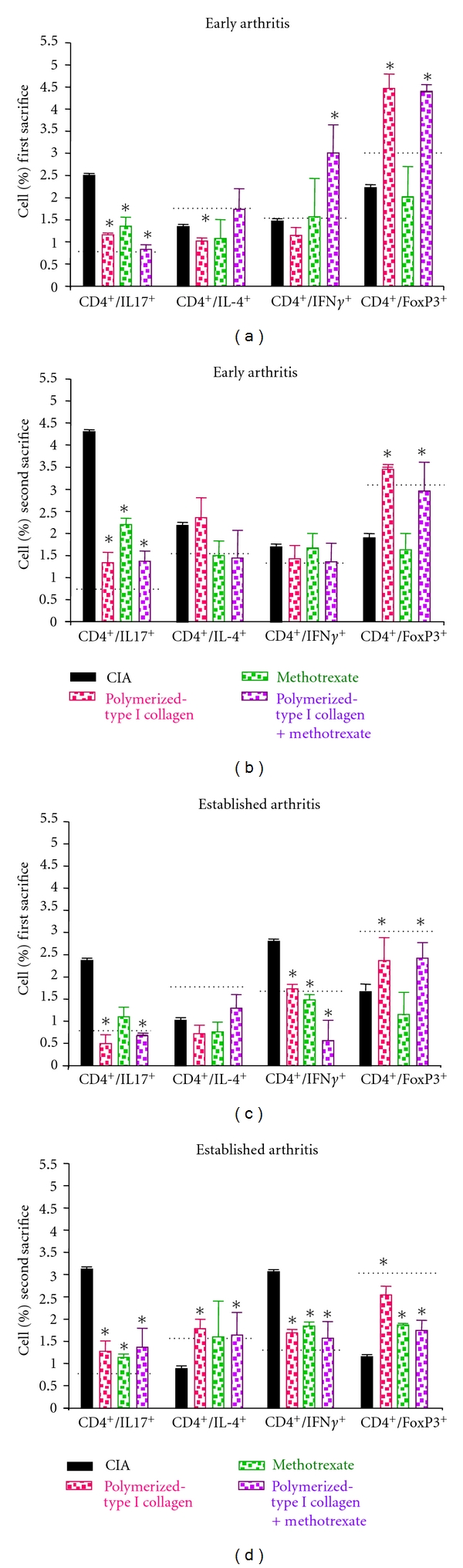
Effect of polymerized-type I collagen on the *ex vivo* intracellular cytokine production and on CD4^+^ T cell subsets regulation in splenocytes. (a) Spleen cells obtained immediately *ex vivo *in early arthritis model on day 35 after booster immunization. (b) Splenocytes obtained immediately *ex vivo* in early arthritis model on day 98 after booster immunization. (c) Spleen cells obtained immediately *ex vivo* in established arthritis model during first sacrifice. (d) Splenocytes obtained from established arthritis model during second sacrifice. Intracellular production of IL-17A, IL-4, IFN-*γ*, and Foxp3 by CD4^+^ T cells was detected by flow cytometry. Results are representative of 6 mice analyzed in each group. Horizontal dotted line represents mean normal values, obtained from mice (*n* = 3) without CIA. Data represent mean ± SEM. *P < 0.05.

Proportions of Th17 were consistently *∼*0.7% in splenocytes from mice without CIA. Higher percentage of IL-17-producing CD4^+^ T cells were determined in CIA mice in early arthritis (2.5% and 4.3%, for the first and second sacrifice, resp. Figures 4(a) and 4(b)) and established arthritis (2.4% and 3.6%, for the first and second sacrifice, resp. Figures 4(c) and 4(d)). A reduction on percentage of IL-17A-producing CD4^+^ T cells similar to normal levels was determined in mice with early and established arthritis treated with polymerized-type I collagen (1.2% and 1.3% for the first and second sacrifice in early arthritis, Figures 4(a) and 4(b) and 0.5% and 1.3% for the first and second sacrifice in established arthritis Figures 4(c) and 4(d)) and methotrexate/Polymerized Type I Collagen (0.8% and 1.4% for the first and second sacrifice in early arthritis, Figures 4(a) and 4(b) and 0.7% and 1.3% for the first and second sacrifice in established arthritis Figures 4(c) and 4(d)). Methotrexate treatment was not so effective compared to the biodrug and the mixture of it with methotrexate (1.4% and 2.2% for the first and second sacrifice in early arthritis, Figures 4(a) and 4(b) and 1.2% and 1.1% for the first and second sacrifice in established arthritis Figures 4(c) and 4(d)). Polymerized-Type I Collagen and methotrexate/Polymerized Collagen treatments had a sustained effect in early and established arthritis model until the second sacrifice (follow up) on the percentage of IL-17A-producing CD4^+^ T (Figures 4(b) and 4(d)). IFN-*γ*-producing Th1 cells were also induced by CII *in vivo *immunization, and their frequency was similar than that of Th17 in early and established arthritis. However, IFN-*γ* producing Th1 cells were lower after treatment in established arthritis model (Figures 4(c) and 4(d)).

Proportions of Treg cells were consistently *∼*3.0% in splenocytes from mice without CIA. Lower percentage of Treg cells were determined in CIA mice in early arthritis (2.2% and 1.6%, for the first and second sacrifice, resp. Figures 4(a) and 4(b)) and established arthritis (1.5% and 1.2%, for the first and second sacrifice, respectively, Figures 4(c) and 4(d)). A statistically significant increase on percentage of Treg cells was determined in mice with early and established arthritis treated with polymerized-type I collagen (4.5% and 3.5% for the first and second sacrifice in early arthritis, Figures 4(a) and 4(b) and 2.4% and 2.5% for the first and second sacrifice in established arthritis Figures 4(c) and 4(d)) and methotrexate/Polymerized Collagen (4.4% and 3.0% for the first and second sacrifice in early arthritis, Figures 4(a) and 4(b) and 2.4% and 1.7% for the first and second sacrifice in established arthritis Figures 4(c) and 4(d)). Methotrexate-treated mice had a slightly increased response on percentage of Treg cells (2.0% and 1.6% for the first and second sacrifice in early arthritis, Figures 4(a) and 4(b) and 1.2% and 1.8% for the first and second sacrifice in established arthritis Figures 4(c) and 4(d)). However, this subpopulation was reduced in 50% compared to mice without CIA.

Taken together, these results indicate that polymerized-type I collagen is a biological compound that downregulated Th17 and upregulated Treg cell differentiation *in vivo *on early and established arthritis. 

### 3.5. Effect of Polymerized-Type I Collagen on NF-*κ*B on Early and Established CIA Model

**Figure 5:**
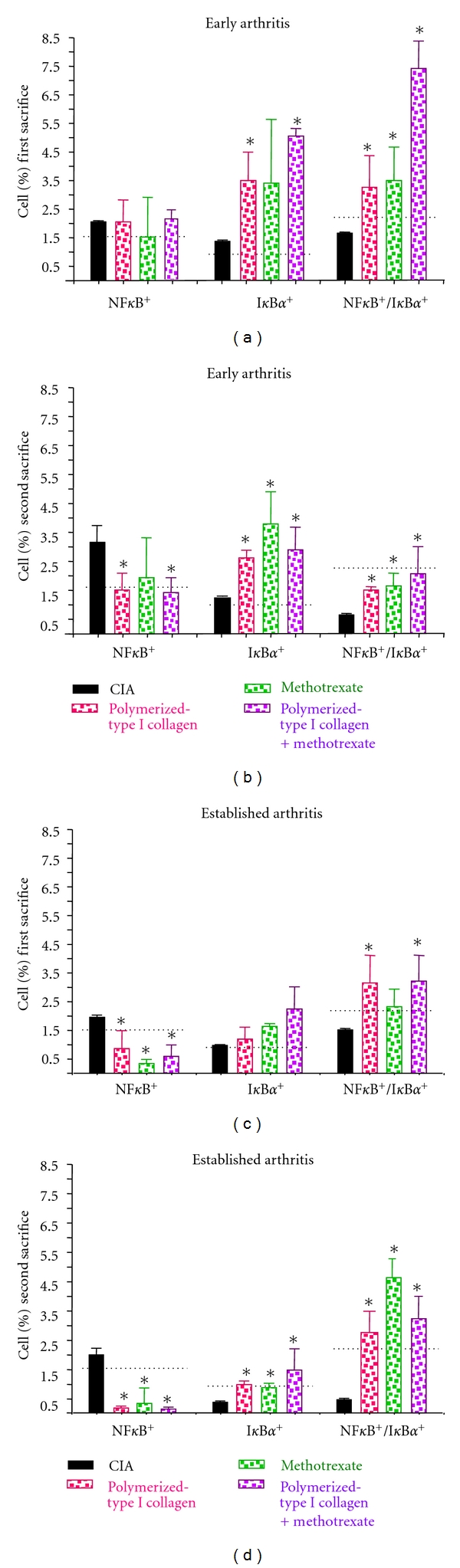
Effect of polymerized-type I collagen on the *ex vivo* NF-*κ*B and I*κ*B-*α* in splenocytes. (a) Spleen cells obtained immediately *ex vivo *in early arthritis model on day 35 after booster immunization. (b) Splenocytes obtained immediately *ex vivo* in early arthritis model on day 98 after booster immunization. (c) Spleen cells obtained immediately *ex vivo* in established arthritis model during first sacrifice. (d) Splenocytes obtained from established arthritis model during second sacrifice. Intracellular levels of NF-*κ*Bp65 and I*κ*B*α* cells were detected by flow cytometry. Results are representative of 6 mice analyzed in each group. Horizontal dotted line represent mean normal values, obtained from mice (*n* = 3) without CIA. Data represent mean ± SEM. *P < 0.05.

We infer that polymerized-type I collagen mechanism of action might be mediated through the regulation of certain transcription factors such as NF-*κ*B and AP-1. In particular, NF-*κ*B regulates the expression of proinflammatory enzymes, cytokines, chemokines, immunoreceptors, and cell adhesion molecules as well as apoptosis. In the light of this knowledge, NF-*κ*Bp65 and I*κ*B*α* were analyzed in splenocytes *ex vivo* (Figure 5). Percentage of NF-*κ*Bp65 (*∼*1.5%), I*κ*B*α* (*∼*1.1%), and NF-*κ*B/I*κ*B*α* cells (*∼*2.2%) were consistently found in splenocytes from mice without CIA.

Higher percentage of I*κ*B*α* ^+^ and NF-*κ*B^+^/I*κ*B*α* ^+^ cells were found in CIA mice under polymerized-type I collagen and methotrexate/polymerized-type I collagen treatment in early arthritis in the first and second sacrifice compared with CIA mice without treatment (Figures 5(a) and 5(b)). Interestingly, normal levels of NF-*κ*Bp65 were observed in CIA mice under Polymerized Collagen and methotrexate/Polymerized Collagen treatment in early arthritis during second sacrifice (Figure 5(b)). In established arthritis model, normal percentages of NF-*κ*Bp65 were found in mice under Polymerized Collagen, methotrexate, or methotrexate/Polymerized Collagen treatment in the first and second sacrifice (Figures 5(c) and 5(d)), as well as, I*κ*B*α* levels during second sacrifice (Figure 5(d)). Finally, NF-*κ*B^+^/I*κ*B*α* ^+^ cells were increased in treated mice compared with CIA mice in the first and second sacrifice (Figures 5(c) and 5(d)).

## 4. Discussion

In the present work, we demonstrated that polymerized-type I collagen as monotherapy as well as in combination with methotrexate exhibits both preventive and therapeutic effects on mouse CIA, through downregulation of Th17 subset and upregulation of Treg cells.

Th17 cells represent a new subset of T helper cells, which mainly produce IL-17A and IL-17F, and, to a lesser extent, TNF-*α* and chemokines. The first report on IL-17-producing CD4^+^ T cells came from a study of *in vitro*-primed TCR transgenic T cells where the addition of *Borrelia burgdorferi* lysate induced IL-17 production [21]. 

However, in the last years, the outstanding importance of Th17 cells has most convincingly been demonstrated in the pathogenesis of organ-specific autoimmune diseases. This was in fact, a broken paradigm, as previously Th1 cells had been regarded as the preponderant cell subpopulation driving autoimmune tissue damage. This concept was challenged when it became clear that IFN-*γ* and IFN-*γ*-receptor deficient mice were not protected from CIA but developed more severe disease [22–26]. Moreover, IL-17-deficient mice or mice treated with anti-IL-17 antibody reduce joint inflammation, cartilage destruction, and bone erosion [27–29]. Collectively, these data corroborate the importance of Th17 cells for the induction of autoimmune tissue inflammation.

Our results are in agreement with the subcutaneous, intramuscular or mucosal administration of type II collagen or altered CII263–272 peptide that suppress Th17 cells and expand regulatory T cells in the early stage of the disease [30–33]. However, the mechanism of action could be quite different meanwhile altered CII263–272 peptides could bind to RA-associated HLA-DR4/1 with no T cell stimulating effects and might inhibit T cell activation in RA, polymerized-type I collagen could be acting likely as a tolerogenic molecule.

Methotrexate therapy is associated with depletion of all CD4^+^ T cell subsets, including Tregs, and also with the development of rheumatoid nodules following improvement of arthritis, as has been reported in previous studies [34–36].

Methotrexate/polymerized-type I collagen induced effective downregulation of inflammatory T cell subsets although, unexpectedly, there was no synergistic effect between both treatments.

On the other hand, it is well known that impaired Treg function is also associated with pathogenesis of autoimmune disease, and that CD4^+^/CD25^+^/Foxp3^+^ cells represent one of the major Treg cells involved in susceptibility/resistance to autoimmunity [37]. In the present experiments, we found that the percentage of Treg cells in the spleen of CIA mouse was significantly upregulated by Polymerized Collagen and methotrexate/polymerized-type I collagen treatments but not by methotrexate alone. These results support the hypothesis that the reciprocal downregulation of IL-17 expression at the time of upregulation of Foxp3 induced by Polymerized Collagen is involved in the balance of Treg/Th17, and this can lead to prevention or full blown autoimmune arthritis.

It is important to note that, 28 days after the last polymerized-type I collagen or methotrexate/polymerized-type I collagen administration, mice were without any sign of disease. Besides, side effects were not determined in mice under Polymerized-Type I Collagen, albeit methotrexate induced other tissue abnormalities such as the presence of nodules (amorphous fibrin tissue like) and hyperthermia (∆≈1.5°C) determined during all the study, which was also previously described by Lange et al. [19].

Furthermore, animals treated with Polymerized Collagen in the early and established CIA model not only remained without clinical manifestations of the disease and without hyperthermia, but their joints were free from inflammatory cell infiltrates. Indeed, histological sections of ankle from polymerized-type I collagen but not methotrexate treatment showed normal joint tissues, without inflammatory infiltration of bone erosions and preservation of proteoglycans content (PAS staining), compared to CIA. Thus, histological evaluations confirmed the characteristic arthritic lesions and showed an excellent correlation with clinical grading. 

NF-*κ*B has been often termed a “central mediator of the immune response,” because of its critical role in the control of key physiological and pathological states, from immune to autoimmune response. Regulation of the so-called “canonical” NF-*κ*B transcription factor by the IKK complex involves its cytosolic-to-nuclear translocation mediated by the phosphorylation of the inhibitory molecule I*κ*B-*α* [38]. Imbalance of NF-*κ*B and I*κ*B*α* has been associated with development of common inflammatory diseases including ulcerative colitis, Crohn's disease, rheumatoid arthritis, systemic lupus erythematosus, psoriatic arthritis, giant cell arthritis, type 1 diabetes, multiple sclerosis, celiac disease, and Parkinson's disease, as well as susceptibility of several cancers, such as oral squamous cell carcinoma, colorectal cancer, hepatocellular carcinoma, breast cancer, and myeloma. In the present work, we found that Polymerized Collagen alone or in combination with methotrexate is capable to increase I*κ*B-*α* inhibitor in early and established arthritis models. Thus, it is not preposterous to speculate that, this biodrug could contribute considerably to the downmodulation of inflammation and tissue regeneration effects observed.

Summing up, polymerized-type I collagen may be one of the novel therapeutic candidates that can suppress autoimmune inflammation by regulating the T cell differentiation and the balance of pathogenic and regulatory T cells, in such a way that proinflammatory Th17 cells are downregulated and Treg cells are expanded. Besides, Polymerized Collagen induces downregulation of proinflammatory cytokine expression and tissue regeneration that could be regulated probably through NF-*κ*B modulation. Our results shed further light into the preponderant role of polymerized-type I collagen in downregulation of inflammation and tissue regeneration and certainly deserve to be studied in depth in order to determine the precise mechanism(s) of action.
